# Nano Copper Oxide-Modified Carbon Cloth as Cathode for a Two-Chamber Microbial Fuel Cell

**DOI:** 10.3390/nano6120238

**Published:** 2016-12-09

**Authors:** Feng Dong, Peng Zhang, Kexun Li, Xianhua Liu, Pingping Zhang

**Affiliations:** 1 Tianjin Key Laboratory of Indoor Air Environmental Quality Control, School of Environmental Science and Engineering, Tianjin University, Tianjin 300072, China; dongfengtju@foxmail.com (F.D.); da.feipeng@163.com (P.Z.); 2 School of Environmental Science and Engineering, Nankai University, Tianjin 300071, China; likx@nankai.edu.cn; 3 College of Food Science and Engineering, Tianjin Agricultural University, Tianjin 300384, China

**Keywords:** microbial fuel cells, nanocatalyst, electrodeposition, cathode, copper

## Abstract

In this work, Cu_2_O nanoparticles were deposited on a carbon cloth cathode using a facile electrochemical method. The morphology of the modified cathode, which was characterized by scanning electron microscopy (SEM) and Brunauer-Emmett-Teller (BET) tests, showed that the porosity and specific surface area of the cathode improved with longer deposition times. X-ray photoelectron spectroscopy (XPS) and cyclic voltammetry (CV) results showed that cupric oxide and cuprous oxide coexisted on the carbon cloth, which improved the electrochemical activity of cathode. The cathode with a deposition time of 100 s showed the best performance, with a power density twice that of bare carbon cloth. Linear sweep voltammetry (LSV) and electrochemical impedance spectroscopy (EIS) results revealed that moderate deposition of nano copper oxide on carbon cloth could dramatically reduce the charge transfer resistance, which contributed to the enhanced electrochemical performance. The mediation mechanism of copper oxide nanocatalyst was illustrated by the fact that the recycled conversion between cupric oxide and cuprous oxide accelerated the electron transfer efficiency on the cathode.

## 1. Introduction

Microbial fuel cells (MFCs) harness the power of a microorganism and convert energy released in metabolic reactions into electrical energy. This environmentally-friendly process produces electricity without the combustion of fossil fuels. Catalysts used to fabricate MFC cathodes are mostly noble metals, such as platinum (Pt). However, the high cost of noble metals inhibits their use in large-scale production of MFCs [1].

Copper is considered to be an important element for cathode catalysis in fuel cells due to its low cost and high stability. Previous studies have demonstrated that the electron-catalytic activity of copper deposited on a carbon cathode for oxygen reduction reaction was fairly good at low oxygen pressure [2]. Recently, it has also been found that Cu_2_O exhibited excellent electron-catalytic activity for the reduction of oxygen. Cathodes decorated by alloys containing copper and other elements, such as Pt, Pd, Mn, and Ir, were developed to replace costly platinum cathodes and showed high power output [3,4,5,6]. In addition, a Pt cathode modified by copper oxide was reported to have the potential for oxygen reduction, and the oxidation state of Cu enables the Cu to act as an adsorption site of oxygen [7]. In addition to oxygen reduction, nitrates and nitrites can be electrochemically reduced by the catalytic activity of copper. Other metals, such as nickel [8], tin [9], zinc [10,11], palladium [12], and rhodium [13] have the same effect. Even carbon monoxide and carbon dioxide have been reported to be the objects of electrocatalytic reduction using cathodes decorated by copper-containing material [14] or copper electrodes [15]. The above-mentioned research indicated the extensive and profound usefulness of copper for improving the efficiency of substrate reduction at the cathode.

However, there is little research about the performance and mechanism of treated carbon cloth by electrodeposited copper in MFC. In this work, a carbon cloth cathode modified by nano copper oxide was prepared using a simple electrochemical method. The effects of deposition time on morphology and catalytic activity of copper oxide were also examined. MFC with potassium ferricyanide as an electron acceptor has high voltage, low internal resistance, and good stability, and it is suited for research purposes [16,17,18]. Baker’s yeast, *Saccharomyces cerevisiae*, has been studied as a biocatalyst for use in biofuel cells [19,20,21], because it has many attractive features, i.e., nonpathogenic, inexpensive, easy mass cultivation, and can be maintained for a long time in a dried state. Therefore, we utilized potassium ferricyanide as an electron acceptor and yeast as a biocatalyst in this study.

## 2. Results and Discussion

### 2.1. Scanning Electron Microscopy (SEM) and Brunauer-Emmett-Teller (BET) Characterization

**Figure 1:**
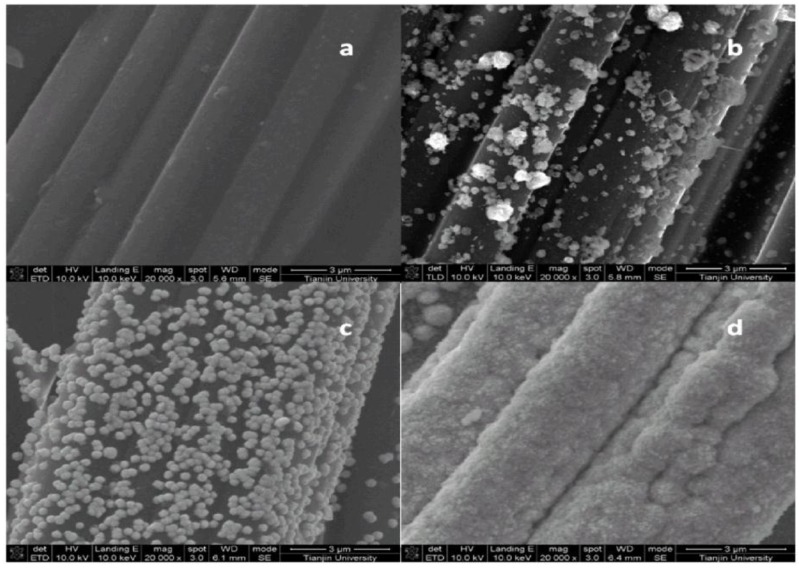
Scanning electron microscopy (SEM) images of the surface of copper oxide-coated carbon cloth. These carbon cloths were modified with deposition times of 0 (**a**), 50 (**b**), 100 (**c**), and 150 s (**d**), respectively.

Figure 1 showed the size of copper oxide particles on the surface of the carbon cloth increased as the deposition time increased. No Cu_2_O particles were found on the bare carbon cloth. The particles with deposition time of 50 s were irregular in shape and sizes and were distributed normally with a diameter of approximately 100 nm, whereas more homogeneous and larger sized (approximately 200 nm) particles were found with a deposition time of 100 s. As the Cu deposition time increased, the size of the particles increased, and the spaces between them became smaller, leading to smaller specific surface areas. As shown in Figure 1, particles with a deposition time of 150 s stood ‘shoulder to shoulder’, which decreased the porosity and the specific surface area of the modified electrode. The corresponding Brunauer-Emmett-Teller (BET) results showed that the specific surface area of the modified electrodes, with deposition times of 50, 100 and 150 s, were 0.1630, 0.2841 and 0.2673 m^2^/g, respectively, indicating that the specific surface area of the cathode improved with longer deposition times, but excessive deposition time led to decreasing specific surface area.

### 2.2. X-ray Photoelectron Spectroscopy Results

**Figure 2:**
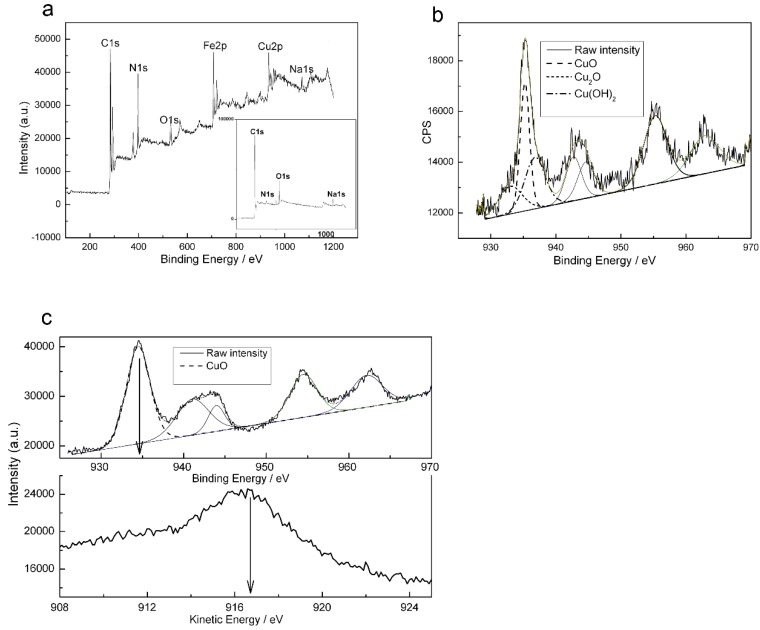
X-ray photoelectron spectroscopy (XPS) spectra of the cathode materials. (**a**) Wide-scan XPS survey spectra of the modified carbon cloth after being applied as cathode in the microbial fuel cell (MFC) for a period of time. Inset: bare carbon cloth. (**b**) Cu 2p core-level. (**c**) The XPS spectrum of the unused copper-coated carbon cloth; the upper and lower part were the spectra of Cu 2p and Cu LMM (L-inner level-M-inner level-M-inner level electron transition), respectively. This material had been dried at room temperature for 24 h.

X-ray photoelectron spectroscopy (XPS) was used to determine the state of the copper on the cathode. Wide-scan XPS survey spectra were obtained from the modified carbon cloth (Figure 2a) and the bare carbon cloth (Figure 2a inset), and the modified carbon cloth was applied as cathode in the MFC for a period of time. The spectra showed that Cu and Fe appeared on the modified carbon cloth in addition to C, O and N and the distributions are shown in Table 1. The presence of Fe and the increase in N on the modified carbon cloth may be attributed to adsorption on the cathode of the ferricyanide in the solution. This result is supported by the measured ratio of Fe to N on the modified carbon cloth being 5.14%:30.13%, which is similar to the ratio in ferricyanide (1:6). Figure 2b shows the core-level Cu 2p region of an XPS spectrum acquired from the modified carbon cloth and a curve-fit to the spectrum. Spectrum noise was attributed to the short acquisition time that was used to avoid the photo-reduction of Cu(II) ions by the action of X-rays [22]. The presence of the well-known shake-up peak indicated the presence of Cu(II) species [23]. Other states of Cu are also present, based on the wide shake-up peak type [24,25]. The large asymmetric mean peak could be decomposed into three contributions at 933.00 eV, 934.7 eV and 935.78 eV (Table 1). The contributions at 934.7 eV and 935.78 eV, according to the literature [23], are due to CuO and Cu(OH)_2_ [26,27], respectively. The first peak at 933.00 eV cannot be specifically identified due to the presence of statistically similar binding energy (BE) values for Cu metal and Cu (I) oxide species. Auger peak position has been used to identify the oxidation state of copper. Previous reports [28,29] had noted a distinctive Cu LMM (L-inner level-M-inner level-M-inner level electron transition) peak at 569.99 eV for Cu_2_O, which is in accord with the Cu LMM Auger peak position of this result (Cu LMM peak at 569.89 eV). Therefore, based on the observed oxidative states of copper, our results indicate that Cu(I) and Cu(II) coexist on the cathode. Thus, the major components deposited on the carbon cloth electrode were Cu in different oxidative states.

XPS spectra, showing Cu 2p and Cu LMM regions, from the electrodeposited carbon cloth that had not been used in the MFC were showed in Figure 2c. The material was dried in a holder at room temperature for 24 h. The figure showed characteristics typical of CuO: The binding energy of Cu 2p being 934.7 eV; the strong shake-up peak of the Cu 2p spectrum; and he peak kinetic energy of Cu LMM spectrum being 916.8 eV. So CuO alone was present on the carbon cloth after electrodeposition. Cu(OH)_2_ was not found on this electrodeposited carbon cloth, and this may be caused by the long drying process, during which the Cu(OH)_2_ converted to CuO.

The following mechanism has been proposed for the formation of CuO on the carbon cloth [30].

Electrodeposition of Cu:

Cu^2+^ + 2e^−^ → Cu, (1)

Formation of Cu(OH)_2_:

Cu + 2OH^−^ → Cu(OH)_2_ + 2e^−^, (2)

Formation of CuO:

Cu(OH)_2_ → CuO + H_2_O, (3)

The proposed mechanism suggests that there is initial formation of Cu(OH)_2_ which then converts into CuO. However, as mentioned above, CuO and Cu_2_O coexist on the cathode after this carbon cloth was applied in the MFC for a period of time. The conversion between the two oxidation states of copper on the carbon cloth cathode may initiate different electrochemical performance which is displayed next.

### 2.3. Performance of MFCs Equipped with the Modified Carbon Cloth Cathodes

**Figure 3:**
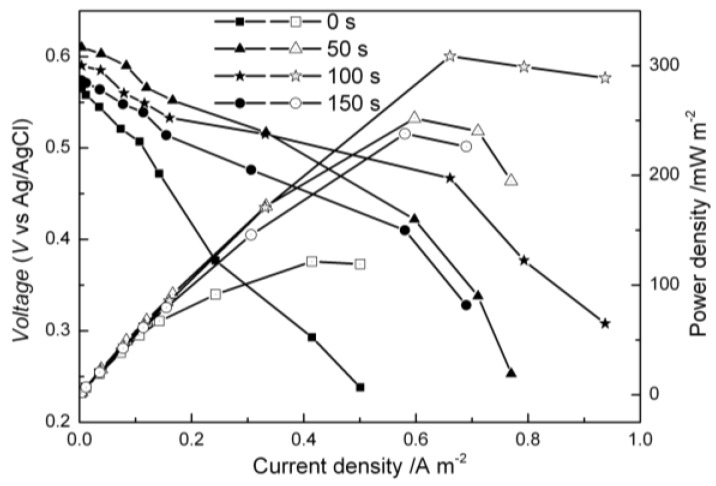
Power densities of four microbial fuel cells (MFCs) with different deposition times.

Tests of MFCs with the modified cathodes were conducted to determine the influence of the deposition on the MFC power generation. Upon variation of the deposition time, the power density curves were also obtained (Figure 3).

As shown in Figure 3, The open circuit voltages (OCV) observed here were approximately 600 mV, which were higher than the values in a previous report [31], but were in accord with Hubenova’s research [32]. The maximum power density of the MFC with a deposition time of 100 s was found to be 308.69 mW/m^2^, over two times higher than that of the bare cathode (121.51 mW/m^2^). The maximum power density of the bare cathode was on the same level as other reports [32,33]. MFCs of modified cathodes with deposition times of 50 s (252 mW/m^2^) and 150 s (237 mW/m^2^) also displayed higher power density than the bare carbon cloth cathode did.

From slopes of the polarization curves in Figure 3, the internal resistances (*R_int_*) of the cell were estimated: 948.88 Ω (0 s), 585.81 Ω (50 s), 374.15 Ω (100 s), and 464.07 Ω (150 s). The *R_int_* of MFCs with modified cathodes were significantly lower than that of the bare cathode, and the *R_int_* of the MFC with a deposition time of 100 s was less than half of the bare cathode MFC’s *R_int_*. This reduction in *R_int_* may be the result of catalysis by the deposited material. The better performance of the modified carbon cloth also found expression in a higher short circuit current density of 0.94 A/m^2^ in comparison with 0.50 A/m^2^ for the bare carbon cloth.

These results clearly showed that modification of the carbon cloth could enhance the MFC power output performance, which corresponded with catalysis by copper [34,35]. The best performance was observed with the deposition time of 100 s. This phenomenon was due to the electroactivity being affected by deposition time [30].

### 2.4. Electrochemical Characterization of Cu Oxide-Coated Carbon Cloth Cathode

**Figure 4:**
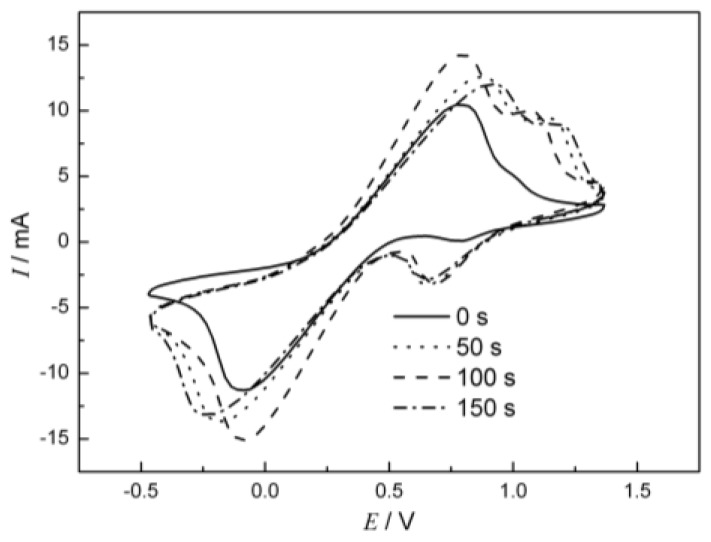
Cyclic voltammetry (CV) of copper-coated carbon cloths. These carbon cloths had deposition times of 0, 50, 100 and 150 s, respectively.

To provide further insight into the role of coated Cu oxide in the catalysis process, different electrochemical characterizations were conducted. Figure 4 showed the cyclic voltammograms (CVs) of the Cu oxide-coated carbon cloths with different deposition times in a potassium ferricyanide solution (50 mM). The peaks of one distinct redox couple with potentials of about −0.1 V and 0.6 V are clearly seen both for the modified carbon cloths and the bare carbon cloth. These redox peaks are related to the oxidation and reduction of ferrocyanide and ferricyanide at the surface of the carbon cloth [36]. In contrast, another redox couple was found with potentials of approximately 0.6 V and 1 V only for the modified carbon cloths. These results show that there is another redox couple that occurs on the modified carbon cloths besides the ferrocyanide and ferricyanide redox. This is consistent with the XPS results that indicate that there are only two oxidative states of Cu present, corresponding to this redox couple. This reductive peak is due to the reduction of CuO to Cu_2_O on the surface of the electrode, which is easier than the reduction of ferricyanide, whose reductive potential was more negative. A similar result had been reported before [35], with CuO nanoparticles playing a role in the increase of the electroactive surface area, but the mechanism had not been explained.

Theoretically, in a potassium ferricyanide solution with high oxidative ability, the Cu_2_O could be easily oxidized to form CuO [30]:

2CuO + 2H^+^+ 2e^−^ → Cu_2_O + H_2_O, (4)

[Fe(CN)_6_]_3_^−^ + e^−^ → [Fe(CN)_6_]_4_^−^, (5)

According to equations 4 and 5, the total reaction was:

2[Fe(CN)_6_]_3_^−^ + Cu_2_O + H_2_O → 2[Fe(CN)_6_]_4_^−^ + 2CuO + 2H^+^, (6)

**Figure 5:**
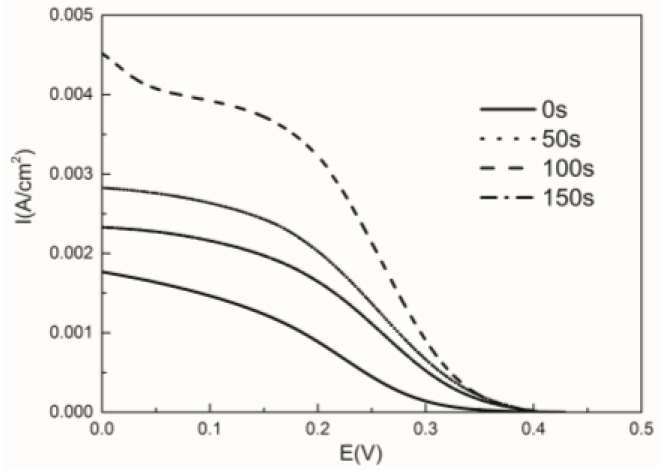
Linear sweep voltammetry (LSV) curves of the bare carbon cloth and the Cu oxide-coated carbon cloths with different deposition times in a 50 mM potassium ferricyanide solution.

Figure 5 shows the linear sweep voltammetry (LSV) polarization curves of Cu oxide-coated cathodes. The slopes of the cathode voltage–current densities are different with the increasing order of 0 s (bare carbon cloth) < 50 s < 150 s < 100 s. The polarization degree of the bare carbon cloth cathode is much greater than that of Cu oxide-coated carbon cloths, indicating that the Cu oxide-coated cathodes have better electrochemical property, which is consistent with the CV results.

**Figure 6:**
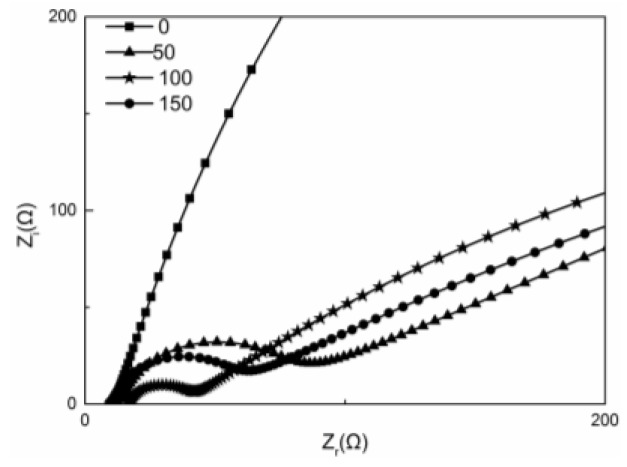
Nyquist plots of electrochemical impedance spectroscopy (EIS) by the Cu oxide-coated carbon cloths with different deposition times.

Figure 6 shows the Nyquist plot for Cu oxide-coated carbon cloths. The total resistance includes the ohmic resistance (*R*_o_), charge transfer resistance (*R*_ct_) and diffusion resistance (*R*_d_). *R*_ct_ is the major kinetic limitation due to slow activation reaction rates on the cathode. *R*_ct_ of the cathodes have an apparent trend, with the order of: 0 s (bare carbon cloth) > 50 s > 150 s >100 s. The *R*_ct_ of bare carbon cloth was 1309 Ω, which was 59.23 times as much as that of cathode with deposition time of 100 s (22.10 Ω) (Table 2).

Cu oxide deposited on the surface of carbon cloth can enhance the electron transfer rate between the current collector and ferricyanide, leading to the decrease of charge transfer resistance. Hence, *R*_ct_ decreases with the increase in deposition time from 0 s to 100 s. However, too much coating of Cu oxide may reduce the specific surface area (Figure 1) and leads to decreased mass transport, which will reduce the cell performance. That is the reason why an optimum deposition time of 100 s exists. These results are consistent with those of CV and LSV tests.

### 2.5. Catalytic Mechanism of Copper Oxide Nanocatalyst

**Figure 7:**
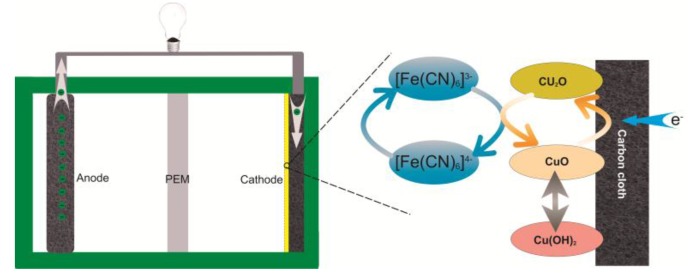
The mechanism of Cu oxide’s intermediation functions on the cathode in ferricyanide solution. The CuO on the cathode surface is first reduced to Cu_2_O by the electrons from the anode, and then Cu_2_O is oxidized to CuO by the ferricyanide. This redox cycle can accelerate the charge transfer rate on the cathode surface.

As mentioned before, the CuO on the MFC cathode is first reduced to Cu_2_O by the electrons from the anode; and then, Cu_2_O is oxidized to CuO by the ferricyanide, and this constitutes a redox cycle mediated on the cathode [37,38]. A schematic diagram of the mechanism is shown in Figure 7. Therefore, the improvement in performance of the MFCs may be the result of the mediation mechanism associated with the Cu oxide film interacting with the ferricyanide solution. It has been reported that copper behaves as a charge mediator for electrocatalytic reduction of nitrite species under neutral conditions [39], which is the same mechanism as has been presented in this paper.

The MFC performances and electrochemical characterization results indicate that the Cu oxide-coated carbon cloth with an intermediate deposition time (100 s) had the highest electrochemical activity. Patake et al. [30] have reported that the electrochemical activity of copper oxide increases with increasing deposition time and with increasing film thickness. The Cu oxide film with a deposition time of 100 s led to better MFC performance, corresponding to reduced charge transfer resistance and increased porosity of surface structure. These results are similar to those observed by other researchers [40]. Thus, improvement of the MFC performance is attributed to the enhanced kinetics and the porosity of the copper oxide film on the electrode.

It should also be mentioned that BET results were not completely consistent with the maximum power densities. Although 100 s and 150 s have almost same surface area, their peak power densities are different, which indicated that the specific surface was not the only factor affecting the performance of MFC. There should be other factors such as the impact of the quantity of deposited copper on carbon cloth, the catalytic activity of copper oxide, and so on. Although the electrochemical characterization and cell performance evaluation of the nano Cu oxide-modified cathodes have been conducted in this work, some other issues, such as long-term stability and use of different electron acceptors, should also be considered in further study in order to advance its practical application.

## 3. Materials and Methods

### 3.1. Electrode Materials and Chemicals

Carbon cloth was employed as the cathode material. The carbon cloth was purchased from Heshen, Inc. (Shanghai, China). Modified carbon cloth was prepared by electrodeposition using a potentiostatic plating technique. The surface area of the carbon cloth was 1 cm^2^. It was hooked onto the titanium wire and then immersed into the electrolyte used for electrodeposition, which contained CuSO_4_ (50 mM), sodium citrate (5 mM), and glucose (20 mM). All chemicals were of analytical grade and were used without further purification. The electrodeposition was performed in a conventional three-electrode cell with platinum wire as a counter electrode and Ag/AgCl as a reference electrode using a Corrtest CS120 model electrochemical workstation (Wuhan, China). The electroplating bath temperature was kept constant at 25 °C by thermostat. The polarization potential of the applied potentiostatic regimes was −0.65 V [41].

### 3.2. MFC Construction and Operation

The catalytic effects of the electrodeposited carbon cloth cathodes were investigated by using them as cathodes in a double-chamber mediatorless yeast-biofuel cell. The cathodic electrolyte was prepared using 50 mM potassium ferricyanide in phosphate buffer solution (PBS) and refreshed every three days. To prepare the PBS, we dissolved 0.27 g KH_2_PO_4_, 1.42 g Na_2_HPO_4_, 8 g NaCl and 0.2 g KCl in 800 mL of H_2_O. Then adjusted the pH to 7.4 with HCl, and added H_2_O until the final volume was 1.00 L. The anode was a round carbon felt with a diameter of 3 cm. Titanium wire was used to connect both the anode and the cathode to the external circuit in order to avoid corrosion of wires due to the electrolyte. The electrodes were fixed in the corresponding anodic and cathodic plastic chamber, each with a volume of 14 cm^3^, and separated from the other by a proton-exchange membrane. The carbon cloth that was to be tested was hooked onto the titanium wire and then immersed into the ferricyanide solution.

*Saccharomyces cerevisiae* was cultivated in yeast peptone medium (YP medium) in an incubator at 30 °C for 16 h to increase the cell biomass. The YP medium was composed of 0.5% yeast extract, 0.8% peptone and 1% glucose, and the pH was maintained at approximately 7 by dissolving these substrates in PBS. After 16 h of cultivation, the medium was injected into the anode chamber, with the biomass of the microorganism having reached a maximum value according to a previous test. Fed-batch MFC operation was performed with a constant load resistance (1 K) as external resistance. After 24 h of operation, half of the anolyte was substituted with yeast-free YP medium.

### 3.3. Electrochemical and Physical Measurements

CVs were measured using a three-electrode conventional cell with the Corrtest CS120 model electrochemical workstation [42]. LSV and EIS were conducted on a CHI 660E electrochemical workstation (Shanghai Chen Hua Instrument Co., Ltd., Shanghai, China). The carbon cloth cathode, Pt electrode, and Ag/AgCl electrode (0.207 V vs. SHE) were used as working, counter, and reference electrode, respectively. CV was measured at a scan rate of 2 mV/s. The current density was calculated based on the cathode area in accordance with Ohm’s law. LSV was measured from the open circuit potential (OCP) to 0 V at a scan rate of 0.1 mV·s^−1^. EIS test was conducted at a frequency range of 100 kHz to 10 mHz with amplitude of 5 mV.

Power density measurements were made using a resistance box at various resistances (10 K to 100 Ω). The cell voltage was recorded using a multimeter [32]. Power density was estimated for each voltage-current density pair on the basis of Joule’s law *P* = *U* × *I* and the corresponding power density curves were also drawn as power density vs. current density plots.

XPS (Axis Ultra DLD, Shimadzu, Kyoto, Japan) was used to evaluate elemental compositions and the chemical states of the copper. The analyzed area of the sample was approximately 0.25 cm^2^. An overview spectrum (0–1200 eV) and narrow regions corresponding to Cu 2p (928–990 eV) were recorded. Deconvolution of the main Cu 2p peaks was performed using Casa XPS software. The cathode material, which was used in the MFC, was removed and washed with distilled water for the test. In addition, electrodeposited carbon cloth that had not been used in the MFC was also tested.

Scanning electron microscopy (SEM, Nanosem 430, FEI, Eindhoven, Netherlands) was used to analyze the morphology of the modified and the bare carbon cloth surface. 5 × 20 mm pieces of the carbon cloth were attached to a piece of glass by conductive tape and were prepared for SEM analysis. Magnifications of 20000× and 100000× were used to inspect the state of the entire film and individual particles, respectively. Specific surface area determination was performed using the BET method with an adsorption meter (ASAP 2020/Tristar 3000, MICROMERITICS, Norcross, GA, USA).

## 4. Conclusions

In summary, copper oxide-coated carbon cloth, which had porous surfaces and an intermediation function, was used for cathodes in MFCs with ferricyanide as an electron acceptor. Cu oxide-coating improved the performance of carbon cloth cathodes. The maximum power density of the MFC with copper oxide-coated carbon cloth with 100 s of deposition reached 308.69 mW/m^2^, more than doubled when compared with bare carbon cloth of 121.51 mW/m^2^. The copper oxide is first reduced by the electrons from the anode to cuprous oxide in the microbial fuel cell, then the cuprous oxide is oxidized to copper oxide by potassium ferricyanide, which forms a redox cycle in the cathode. This redox cycle actually improved the performance of MFC. The improved performance was mainly attributed to the porous surface of the cathode and to the fast and reversible redox reactions between the oxidation states of Cu(II) and Cu(I). With the prolongation of the deposition time, the mass of the deposited copper oxide and the specific surface area of the electrode gradually increased. However, too long of a deposition time leads to the decrease of specific surface area, which reduced the electrocatalytic performance of the electrode and the performance of the fuel cell. This phenomenon was observed for the first time and the corresponding mechanism was identified. This work can help to improve our understanding of the reduction mechanisms of catalytic agents on cathodes.

## Figures and Tables

**Table 1:** Assignments of main spectral bands for carbon cloth and modified carbon cloth. The bands are based on their binding energies (BE) and atomic concentration (AC).

Element	Bare Carbon Cloth		Modified Carbon Cloth	
	BE (eV)	AC (%)	BE (eV)	AC (%)
O 1s	532	11.58	532	10.15
C 1s	284	85.30	284	41.54
N 1s	399	1.97	398	30.13
Na 1s	1071	1.15	1071	1.48
Fe 2p			708	5.14
Cu 2p			933.00	2.64
Cu 2p			934.70	4.75
Cu 2p			935.78	4.18
Total Cu				11.57
Cu LMM (L-inner level-M-inner level-M-inner level electron transition)			569.89	

**Table 2:** Electrochemical impedance fitting results of different cathodes.

Samples	0 s	50 s	100 s	150 s
*R*_o_ (Ω)	10.74	11.38	10.41	8.533
*R*_ct_ (Ω)	1309	84.36	25.72	37.53
*R*_d_ (Ω)	1145	1140	932.5	1171

## Abbreviations

The following abbreviations are used in this manuscript:

MFC: microbial fuel cell
SEM: scanning electron microscopy
BET: Brunauer-Emmett-Teller
XPS: X-ray photoelectron spectroscopy
CV: cyclic voltammetry
LSV: linear sweep voltammetry
EIS: electrochemical impedance spectroscopy
OCV: open circuit voltage
